# Gene Composer: database software for protein construct design, codon engineering, and gene synthesis

**DOI:** 10.1186/1472-6750-9-36

**Published:** 2009-04-21

**Authors:** Don Lorimer, Amy Raymond, John Walchli, Mark Mixon, Adrienne Barrow, Ellen Wallace, Rena Grice, Alex Burgin, Lance Stewart

**Affiliations:** 1 deCODE biostructures, Inc 7869 NE Day Road West, Bainbridge Island, WA, 98110, USA; 2 Seattle Structural Genomics Center for Infectious Disease, Bainbridge Island, WA, 98110, USA; 3 Accelerated Technologies Center for Gene to 3D Structure, Bainbridge Island, WA, 98110, USA; 4 Emerald BioSystems, Inc 7869 NE Day Road West, Bainbridge Island, WA, 98110, USA

## Abstract

**Background:**

To improve efficiency in high throughput protein structure determination, we have developed a database software package, Gene Composer, which facilitates the information-rich design of protein constructs and their codon engineered synthetic gene sequences. With its modular workflow design and numerous graphical user interfaces, Gene Composer enables researchers to perform all common bio-informatics steps used in modern structure guided protein engineering and synthetic gene engineering.

**Results:**

An interactive **Alignment Viewer **allows the researcher to simultaneously visualize sequence conservation in the context of known protein secondary structure, ligand contacts, water contacts, crystal contacts, B-factors, solvent accessible area, residue property type and several other useful property views. The **Construct Design Module **enables the facile design of novel protein constructs with altered N- and C-termini, internal insertions or deletions, point mutations, and desired affinity tags. The modifications can be combined and permuted into multiple protein constructs, and then virtually cloned *in silico *into defined expression vectors. The **Gene Design Module **uses a protein-to-gene algorithm that automates the back-translation of a protein amino acid sequence into a codon engineered nucleic acid gene sequence according to a selected codon usage table with minimal codon usage threshold, defined G:C% content, and desired sequence features achieved through synonymous codon selection that is optimized for the intended expression system. The gene-to-oligo algorithm of the Gene Design Module plans out all of the required overlapping oligonucleotides and mutagenic primers needed to synthesize the desired gene constructs by PCR, and for physically cloning them into selected vectors by the most popular subcloning strategies.

**Conclusion:**

We present a complete description of Gene Composer functionality, and an efficient PCR-based synthetic gene assembly procedure with mis-match specific endonuclease error correction in combination with PIPE cloning. In a sister manuscript we present data on how Gene Composer designed genes and protein constructs can result in improved protein production for structural studies.

## Background

Large-scale projects in genomic sequencing and protein structure determination are producing enormous quantities of data on the relationships between 2D gene sequence and 3D protein structure. Moreover, such efforts are providing experimental data on success factors at every step in the gene to structure research endeavor. Ideally, this wealth of information should be used in a feedback cycle to facilitate the design and production of genes and protein constructs that are optimized for the successful production of functional protein samples for structural studies. Fundamentally, this goal represents a bioinformatics software challenge. With the goal of improving yield and success rates of heterologous protein production for structural studies, we have developed Gene Composer, a database software package which facilitates the information-rich design of protein constructs and their codon engineered synthetic gene sequences.

The redundancy of the genetic code allows any given protein to be encoded by a very large number of possible synonymous gene sequences. On average, each amino acid can be encoded by approximately three different codons (61 amino acid codons/20 amino acids). For a typical 100 amino acid protein there would be 3^100 ^(~5 × 10^47^) different possible coding sequences. The degeneracy of the genetic code therefore allows the pressures of natural selection to simultaneously influence both DNA and RNA sequence features in addition to protein coding function. DNA sequence elements and folded RNA structures are known to play significant roles in gene expression. As such, the overlapping information contained in a gene sequence can be significantly more complex than coding for a linear amino acid sequence. For example in the tryptophan operon of *E. coli*, the mRNA can fold into one of two mutually exclusive conformations that are a direct consequence of tryptophan availability [1]. These alternate conformations affect mRNA stability and therefore alter the expression of the encoded proteins. It is also well established that codon preferences between species, and often between gene families within a given species, can vary [2,3]. Therefore, some gene sequences may behave better than others in supporting high-level translation for heterologous protein expression. Being able to tailor synthetic gene sequences by codon engineering to favor optimal heterologous expression is a well established strategy for improving heterologous protein expression for structural biology [4].

Given the overlapping nature of information content in gene sequences (DNA, RNA, and protein level) we endeavored to create a database and software tool called Gene Composer which facilitates the design of protein constructs, guided by 2D and 3D information, while the corresponding nucleic acid sequences are engineered for both codon usage and other desired sequence features. Gene Composer also enables the virtual cloning of the designed gene constructs which, depending on user preferences, can be parsed into data files for online ordering of complete genes or overlapping oligonucleotides that can be used for PCR-based gene assembly in any standard molecular biology lab. Gene Composer operates within the Windows^® ^operating system and utilizes a network based SQL server or Access^® ^database that is populated by users as they design genes. This arrangement makes it possible for multiple users to go back after time to design new construct variants that improve on existing designs by inclusion of new sequence or structural information from international genome sequencing and structural genomics efforts. In this report we describe how the synthetic gene design modules of Gene Composer facilitate protein construct engineering for structural studies, codon engineering for heterologous protein production, and oligonucleotide planning for PCR-based gene assembly with mismatch endonuclease error correction.

## Implementation and results

### Gene Composer™ Software

Gene Composer has a modular design to facilitate the work of protein engineers and structural biologists. It combines, within a single database software product, the ability to carry out comparative sequence alignments (***Alignment Viewer***) that facilitates interactive protein construct design with virtual cloning (***Construct Design Module***), followed by codon engineering of novel synthetic gene sequences that are optimized for protein expression in various recombinant systems (***Gene Design Module***). Gene Composer is written in C^++ ^for Windows^® ^operating systems, and runs together with either an Access^® ^or SQL^® ^database.

#### Alignment Viewer

**Figure 1:**
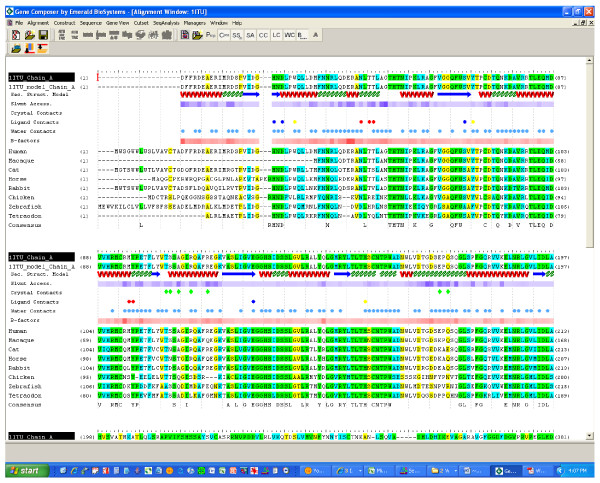
**Gene Composer software, Alignment View**. Comparative sequence alignments and corresponding structural information are organized by the Alignment Viewer of Gene Composer, shown here with PDB file sequences for human renal dipeptidase (1ITU), and the amino acid sequences of full length human renal dipeptidase plus selected vertebrate homologues (FASTA files read from a BLAST search using the amino acid sequence file of PDB file 1ITU). The sequence alignments are produced by the popular ClustalW algorithm [6,7]. The conserved consensus sequence is also shown. Solvent accessible surface area is represented in purple shading levels (light is low and dark is high) calculated according to Connolly [9]. Thermal B-factors from the PDB file are normalized and represented in red shading levels (light is low and dark is high). Residues that contain atoms within 3.5 Angstroms (or other user defined setting) of an oxygen atom of water molecules are illustrated with a blue dot. Residues that contain atoms within 4.0 Angstrom (or other user defined setting) of a non-hydrogen atom of a small molecule ligand are illustrated with colored circles or dots (excluding blue dots for water contacts). Residues that contain non-hydrogen atoms within 4.0 Angstroms (or other user defined setting) of any non-hydrogen atom in a neighboring protein chain are illustrated with a green diamond when space group symmetry operators are applied to build crystallographic neighbor molecules, or a red diamond when the contact is between non-crystallographic symmetrically related polypeptide chains. Protein secondary structural regions are represented by red helices for alpha helical regions, blue arrows for beta-sheet regions, and green squiggles for turns, as defined the respective PDB file format.

A typical gene design cycle is initiated when a user defines a protein "target name" and a "project name" which establishes key database identifiers for which all subsequent Gene Composer workflow is associated. Once these identifiers have been established, the user is presented with a file navigation interface that allows one to import information into the Gene Composer database from multiple sources such as FASTA sequence files from BLAST [5] searches, existing sequence alignments, simple text (.txt) files, and structure files from the Protein Data Bank (PDB, ). From this imported information, Gene Composer uses the popular ClustalW algorithm [6,7] to calculate comparative protein sequence alignments, which are presented in a distilled format within the interactive **Alignment Viewer **(Figure 1). This Alignment Viewer allows the researcher to simultaneously visualize sequence conservation in the context of known protein secondary structure, ligand contacts, water contacts, crystal contacts, residue property type and several other useful property views that are used to guide interactive decision making for protein construct design.

Areas of sequence conservation are highlighted within the Alignment Viewer according to a user defined color scheme and a consensus sequence is displayed below the alignment. Protein secondary structure information is extracted from PDB files and displayed in common graphic annotation underneath their associated linear amino acid sequences. Importantly, the Alignment Viewer presents both the "chain" sequence for the protein that went into crystallization as well as the experimentally refined "model" sequence from the PDB coordinate file. This allows the user to easily visualize which amino acid residues had no structural information reported in the PDB file, displayed as blank gaps in the "model" sequence. Such residues are usually located within highly flexible regions of the protein and do not contribute to X-ray diffraction.

Users can define a threshold contact distance setting (default setting is 3.4 Angstroms) which Gene Composer uses to generate a simple distance matrix between non-bonded, non-hydrogen atom centers in PDB files. The resulting matrix is used to flag residues in the protein model that participate in ligand contacts, water contacts, and/or crystal contacts. Each contact type is annotated within the Alignment Viewer with special visual symbols displayed below the residue of interest (Figure 1). Crystal contacts are indicated when non-hydrogen atoms of a residue are positioned within 4.0 Angstroms of neighboring molecule related by crystallographic or non-crystallographic symmetry. Gene Composer has a database of all protein crystal space groups required for the crystal contact calculation. The ability to visualize residues involved in crystal contacts helps the user to identify residues that could be mutated to improve crystal growth [8]. Gene Composer also calculates from PDB file information the relative solvent accessible Connolly surface area [9] and thermal B-factors for residues which are displayed with relative color intensities to provide a visual representation of the surface location and mobility of amino acids. This facilitates the visual identification of surface residues that are candidates for surface entropy reduction mutagenesis which is a commonly used to aid protein crystallization [10]. The alignments can also be easily modified and annotated with the aid of an interactive cursor that allows the user to insert or delete sequences, residues, or spaces. Importantly, the native amino acid residue numbering scheme is preserved throughout any alignment manipulations. However, since the amino acid sequence numbering scheme of PDB files is often not necessarily congruent with the residue numbering of native full length gene sequences, users can select a given sequence and re-define the starting residue number. In this way, users can arrive at a common residue numbering scheme to help ensure accuracy in subsequent construct design. Finally, the information rich alignments can be saved in the Gene Composer database and exported to several other formats including *.aln, *.xml, and *.pdf for facile data sharing between researchers.

#### Protein Construct Design and Automated Cloning

**Figure 2:**
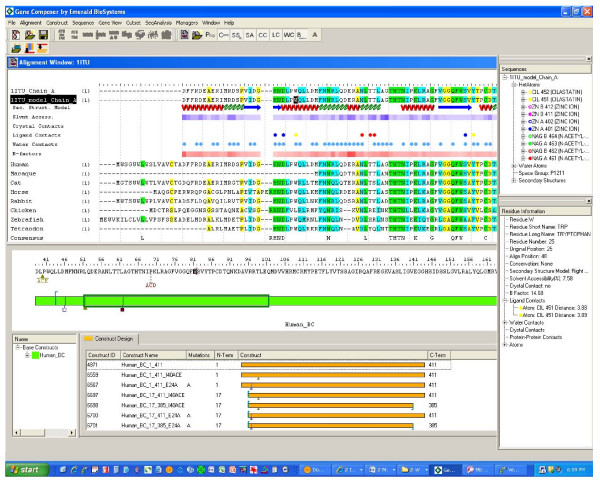
**Gene Composer software, Protein Construct Design**. The Construct Design tool of Gene Composer is a separate window positioned below the Alignment Viewer. To start a Construct Design session, the user defines a Base Construct from the Alignment Viewer (human protein sequence with black highlighted box), whose sequence is presented schematically (green bar) with a sliding window (green box) that delimits the amino acid sequence of the portion of the Base Construct within the sliding window (sequence above the green bar). The Alignment Viewer and Construct Design Tool are coordinated to move with each other as the user conducts various construct design operations across the Base Construct. Desired truncation end points are set by placing the cursor (red line in alignment) at desired sequence positions and inserting a right or left pointer for N-terminal and C-terminal truncations, respectively. The user may also create mutations within the amino acid sequence either as a single amino acid change (empty square), insertions (filled triangle above the inserted amino acids), deletions (filled, inverted triangle), or a mutation pool defining multiple mutations at a single site (dotted line above the desired changes). Once the desired construct modifications have been defined, the user can combine and permute any desired set of truncations and mutations to generate virtual constructs shown in the lower Construct Design window. Each construct is given a unique Construct ID and Construct Name according to a standardized schema that describes the N- and C-terminal residue numbers and other features.

**Figure 3:**
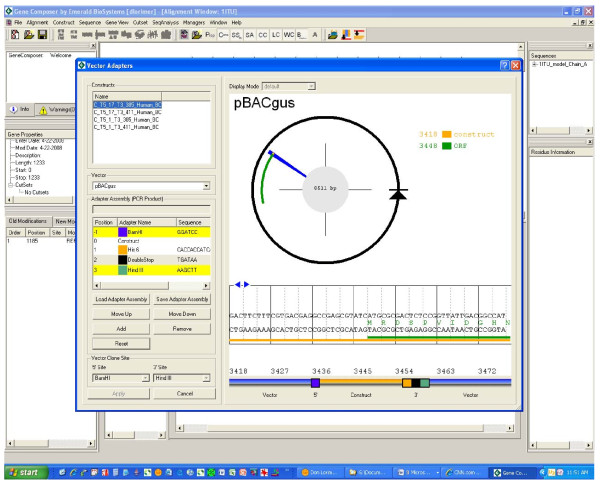
**Gene Composer software, Vector construct assembly viewer**. Virtual construction of expression vectors with designed gene inserts is accomplished through the Vector Construct Assembly Viewer shown here. All vectors, adaptor assemblies, and engineered gene constructs are stored in the Gene Composer database. Virtual cloning starts with the selection of all gene constructs that are to be cloned (upper left, Construct window). The user then selects a desired plasmid expression vector (pBACgus displayed as black circular arrow). Based on data associated with the expression vector, the Gene Composer software is cognizant of the intended point of insertion for the protein open reading frame (green arc). The sequence details are shown with a radial sequence viewer (blue wedge), which displays the nucleic acid sequence in that region (window with blue arrows). User defined adaptor assemblies (Adaptor Assembly window, lower left) are used to combine the selected expression vector with desired gene construct inserts (orange line). The user arranges the adapters in the order desired, selects a vector from the pull-down menu and the software assembles the elements into a final construct. In the example shown, there is a BamHI restriction site (blue square) at the 5' end of the insert, while the 3' end contains an in frame hexa-histidine (His6) amino acid affinity tag sequence (orange square), a double translation terminator (black square), and a HindIII restriction site (green square). The amino acid sequence of the construct plus added tags is also displayed.

The **Construct Design Module **works in concert with the Alignment Viewer allowing the researcher to interactively define novel protein constructs with altered amino- and carboxy-termini, internal insertions or deletions, point mutations, and added affinity tags. The construct design tools are connected to the Alignment Viewer by a cursor that shows the user exactly where in the sequence alignment the desired changes are being made. For example, the user can set the cursor within the Alignment Viewer at a domain boundary as visualized in the comparative sequence alignment and then truncate the construct at that site. The desired modifications can be virtually combined and permuted *in silico *to arrive at multiple desired protein constructs (Figure 2). The user can also add a variety of adaptor assemblies at the DNA sequence level to facilitate the virtual and physical cloning of the constructs into multiple defined expression vectors (Figure 3). Importantly, the Gene Composer virtual *in silico *cloning utility manages the inserts, vectors, and adaptor assemblies as three independent informatics components that are combined by the user to arrive at final vector clones [11]. After the virtual cloning is completed, the user can inspect the entire vector with its adaptor assemblies and protein construct inserts. In this way, the user can see exactly how open reading frames are constructed and then easily fix any virtual cloning errors before wet lab work is performed. Many expression vectors come with their own N-terminal or C-terminal affinity tags that must be accurately fused in frame with the protein construct. Visual inspection of the virtual clone ensures that the open reading frame formed by the vector/adaptor/insert combination is intact and accurate.

In order to automate the physical cloning of designed constructs using either the Tecan Freedom Evo2 liquid handling system  or handheld pipettors, the Construct Design Module can automatically plan out all of the required amplimers (primers) and mutagenic oligonucleotides needed to produce the gene constructs of interest by PCR from a defined template. The template sequence can either be a native cDNA sequence or a codon engineered synthetic DNA sequence crafted by the Gene Design Module of Gene Composer. The software creates a spreadsheet list of the required oligonucleotides with defined quantity and location within a bar coded 96 well plate that conforms to Society of Biomolecular Screening (SBS) standards. The spreadsheet is sent directly to oligonucleotide vendors (e.g. IDT, Inc., Coralville, IA) who will typically prepare and ship the oligo-plates within a week. Once SBS plates containing the amplimers (terminal primers) and mutagenic oligonucleotides have been obtained, they can then be placed on the deck of the Tecan liquid handler together with plates containing template DNA (either cDNA or synthetic DNA), vector DNA, buffer mixes, polymerase, and other enzymes at defined locations. With this information, the Construct Design Module can compute a script of pipetting instructions for the Tecan liquid handler to perform all the necessary liquid handling events needed to pool template DNA with amplimers, polymerase, and buffers within individual wells of a 96-well PCR plate. Alternatively, the same script of pipetting, PCR, and cloning events can be executed manually by a researcher with a handheld pipettor. After the PCR plates are filled with the necessary reagents, either the user or the robotic arm of the Tecan robot places them onto thermal cycler which carries out the necessary PCR reactions to produce "adapted" inserts. In the case where point mutations, deletions, or insertions are required at a single site within the insert sequence, the Tecan platform (or the researcher with a hand held pipette) is scripted to add mutagenic oligos to plasmid constructs with inserts, wherein the QuikChange^® ^site directed mutagenesis method is then used to obtain the desired mutated constructs [12].

Gene Composer supports recombinational cloning (ie. Gateway^®^), restriction enzyme (RE) cloning, ligase independent cloning (LIC) [13], and polymerase incomplete primer extension (PIPE) cloning [14]. For Gateway^® ^cloning, Gene Composer designs terminal amplimers (forward and reverse) with the required recombination sequences recognized by recombinase enzyme to affect recombination with the vector DNA to form the final constructs. For restriction enzyme cloning, Gene Composer allows the user to create custom terminal amplimers with defined terminal restriction enzyme recognition sites and any extra bases needed to ensure efficient restriction enzyme cutting at ends. For LIC and PIPE methods, Gene Composer allows the user to define standard terminal amplimers with the necessary terminal LIC or PIPE cloning sequences.

The Gateway^®^, RE, and LIC methods for cloning each require that the PCR insert samples be subjected to an extra step of treatment or purification prior to mixing with the vector sample for final cloning. In the case of Gateway^® ^cloning, the PCR products must be purified away from amplimers that could otherwise interfere with vector recombination. For RE cloning, the inserts must be digested with restriction enzymes and then purified away from the enzymes and terminal bits of DNA released during digestion. For LIC cloning the inserts must be purified away from nucleotides and primers, so that they can be treated with T4 DNA polymerase to affect a specific 3' to 5' single strand digestion of DNA termini in the presence of a specific a defined deoxynucleotide triphosphate mix [13]. Each of these steps adds extra time, cost, and complexity to the cloning process. Moreover, in cases where the open reading frame of the expressed target protein is produced as a fusion protein encoded in part by sequences within the vector, each of the Gateway^®^, RE, and LIC procedures produces open reading frames with "extra" non-native amino acids encoded by the terminal cloning sequences used to adapt the insert to the vector.

**Figure 4:**
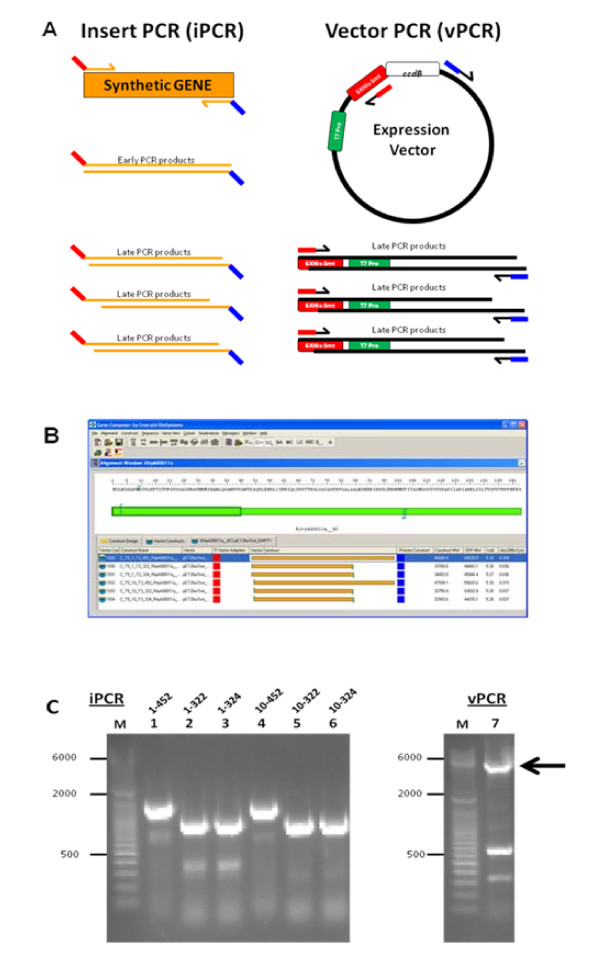
**Amplimer planning for PIPE cloning**. Gene Composer was used to plan the virtual and PIPE cloning of full-length and terminal deletion constructs of a codon engineered *Bacillus subtilis *FtsZ gene. **A**, PIPE cloning is illustrated wherein the synthetic gene insert (orange) is amplified by designed forward (red-orange lines) and reverse (orange-blue lines) primers to generate insert PCR material (iPCR, ~900–1350 bp). The expression vector is amplified with reverse (red-black lines) and forward (blue-black lines) primers to generate vector PCR material (vPCR, full length is ~5.8 Kbp). The terminal sequences iPCR products are complementary to the terminal sequences of vPCR products (red of iPCR complements red of vPCR and blue of iPCR complements blue of vPCR). This allows the iPCR and vPCR products to anneal to form circular DNA molecules that are replicated upon transfection into host bacteria. **B**, Based on alignment analysis of the target amino acid sequence for FtsZ to relevant PDB entries, terminal truncation points were selected (denoted by vertical bars in the base construct view). Permutations of the terminal deletions were used to generate multiple constructs that were virtually cloned according to PIPE parameters in Gene Composer (listed in the vector construct tab, below the base construct view). **C**, Agarose gel analysis of PIPE Cloning constituents. After the terminal deletions of FtsZ-encoding constructs were designed and virtually cloned into the expression vector. Gene Composer generated a spreadsheet file of all necessary amplification primers to produce iPCR and vPCR products. This oligonucleotide file was uploaded directly to the oligonucleotides synthesis vendor website together with information dictating the specific positions of the designed oligos in a 96-well plate. The, lyophilized oligos were resuspended and used directly in an automated PIPE (polymerase incomplete primer extension) cloning procedure on a Tecan EvoFreedom liquid handling robot. Agarose gel analysis of the iPCR in lanes 1–6, and vPCR in lane 7 products are shown together with molecular size standards.

In contrast, PIPE cloning enables the direct enzyme-free cloning of PCR products without any unwanted "extra" amino acid sequences in the final cloned open reading frame. The PIPE method relies on the observation that a significant quantity of PCR products have incompletely polymerized strands, leaving single-stranded 5' overhangs of various length defined by the variable 3' end points of the complementary strands [15]. Since the PCR products all begin at 5'-ends that are defined by the forward and reverse amplimers, both strands of a duplex will together encompass the entire desired coding sequence. With PIPE cloning, one set of primer pairs are used to amplify the cloning vector in one PCR reaction (vPCR), such that it is linearized with 5' overhangs whose ends are complementary to 5' overhangs of insert PCR products (iPCR) which are generated from a second pair of amplimers used to amplify the insert DNA. Mixing the insert and vector PCR products creates stably annealed fragments suitable for direct cloning by transfection into bacteria whose DNA replication and repair machineries effectively produce the plasmids of interest. Virtually any PCR product can be inserted into virtually any vector using this PIPE approach, drastically reducing time and materials required in a high throughput, cloning platform. Gene Composer supports PIPE cloning by planning out all of the required vPCR and iPCR reaction amplimers needed, as well as the scripted pipetting steps to be executed by the Tecan platform to complete the mixing of vPCR and iPCR products so that they are ready for direct transformation of bacteria (Figure 4).

### Gene Design

The **Gene Design Module **uses a protein-to-DNA algorithm to automate the "back-translation" of a protein amino acid sequence into a codon engineered nucleic acid sequence. This can be done for a variety of heterologous expression systems based on their known codon bias represented in pre-installed codon usage tables constructed from the calculated codon frequencies from genomic sequence information [16]. In addition, the Gene Composer database contains modified codon usage tables derived from codon preferences of highly expressed proteins (HEXP-CUT), or hybrid codon usage (HyCUT) designed to accommodate expression in multiple systems, *e.g.*, bacteria or insect cells. Users can also generate their own preferred codon usage tables as more genomic sequence data is published.

Prior to running the back-translation algorithm, users are asked by Gene Composer to select a codon usage table (CUT), define a minimal codon usage frequency threshold to eliminate the use of "rare" codons and set a target guanine:cytosine percentage (G:C%) content for the intended expression system. Codons with fractional usage above the minimal threshold setting are re-normalized so that their fractional usage settings reflect their relative proportional usage when rare codons are eliminated. This allows the back-translation algorithm to plan synthetic "codon engineered" gene sequences that lack rare codons, but otherwise have codon usage which mimics natural codon usage. This is achieved algorithmically by random selection of available codons in a weighted fashion according to their re-normalized fractional usage. Based on these user defined parameters, Gene Composer generates thousands of possible new synonymous gene sequences for any given protein sequence. The software then selects a single synthetic gene sequence that most closely matches the target G:C%.

If users wish to eliminate stable mRNA hairpins from their synthetic gene sequences, the back-translation algorithm can be run in "delta G" mode wherein the standard back-translation is carried out together with a user defined threshold for an allowable calculated change in Gibbs free energy (ΔG) for local RNA folding. In this mode, the standard back-translation algorithm runs the Vienna RNA Package  to calculate local RNA secondary structure and minimum ΔG in a step-wise fashion across the entire gene sequence [17]. The user is allowed to set the window length of the local RNA (typically 50 bases), step size (typically 10 bases) and salt concentration used in the calculation. If the ΔG value for local RNA folding violates a user-defined threshold for stable hairpin formation, then the sequence is rejected and the back-translation begins again. This feature helps to ensure that synthetic codon engineered gene sequences will lack any highly stable mRNA secondary structure such as a hairpin or pseudoknot that could have a negative impact on the fidelity of protein translation [18,19]. The default setting is 100 iterations which the user may adjust to suit his or her preferences. Additionally, the window size and step size can be adjusted. The processing time for this step will vary depending upon the user's equipment. A 3.0 GHz, Pentium 4 processor processed 100 iterations of back-translation in the deltaG mode of p38α (NP_620581, 360 amino acids, 1080 bp of DNA) in approximately 45 sec. This step is only the first step of our process. Detailed below are additional modifications that the user may select to further enhance the gene design and arrive at a highly optimized sequence. We also want the reader to note that the user can elect to skip the back-translation step and begin instead with a native mRNA sequence. When modifying a native mRNA the user still selects a codon usage table and codon frequency cutoff. Within this framework, various engineering tools (listed below) can be applied to modify a native gene sequence. Moreover, the software allows users to visualize graphically the location of rare codons across a gene sequence relative to a selected codon usage table and defined codon frequency threshold. This allows the user to identify rare codons within native gene sequences so that they may be appropriately modified.

#### Synonymous Codon Engineering of Desired Sequence Features

**Figure 5:**
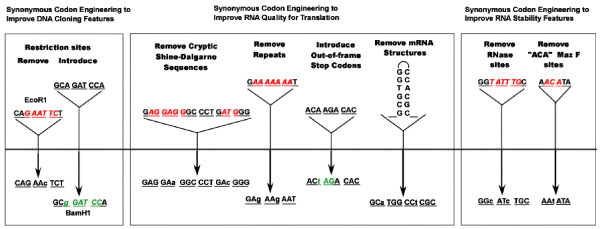
**Use of the degeneracy of the genetic code to silently alter DNA sequences without altering the amino acid sequences**. Synonymous codon changes can be categorized as (i) changes to improve DNA cloning features (left box, removal of an EcoR1 site or introduction of a BamH1 site); (ii) changes to improve RNA quality for translation (middle box, removal of a cryptic Shine-Dalgarno site, removal of base repeats, introduction of "ambush" out of frame stop codons, and removal of mRNA hairpin); and (iii) changes to improve RNA stability (right box, removal of RNase or MazF cleavage sites). The DNA encoding a target gene is shown as a solid black line, with sequences of interest in expanded view. Sequence elements with underlined codon triplets which can be altered are shown above the line and the alternative synonymous sequences are shown below the line.

Beyond simple back-translation, there are at least three general classes of synonymous codon engineering features that synthetic gene engineers may desire: (i) those that facilitate DNA cloning operations such as the introduction or elimination of restriction enzyme cleavage sites; (ii) those that improve the "translation quality" of mRNA such as the elimination of nucleotide repeats, codon repeats, cryptic Shine-Dalgarno translation start sites, or the introduction of alternate reading frame "ambush" stop codons [20]; and (iii) those that improve mRNA stability such as the elimination of RNase cleavage sites or microRNA target sequences [21]. Therefore, the Gene Design Module has been equipped with several unique codon engineering tools that allow users to further modify the back-translated gene sequence with silent/synonymous nucleotide changes to introduce or eliminate nucleic acid sequence elements (Figure 5). Many of the design tool parameters can be set by the user to suit his or her specifications, however all of the design tools have default settings which are used as guides. All of the changes made by the user in later steps are made within the context of the codon usage table and frequency cutoff chosen at the beginning of the design process. When applied to a gene sequence, each of the available codon engineering strategies described below are tracked in the Gene Composer database as a sequence of engineering events. In this way, every step in gene engineering is tracked. Additionally, the user is notified if a modification conflicts with any previous modifications. The user is given the option of accepting or rejecting the modification.

#### Silent Introduction or Removal of Restriction Endonuclease Cleavage Sites

**Figure 6:**
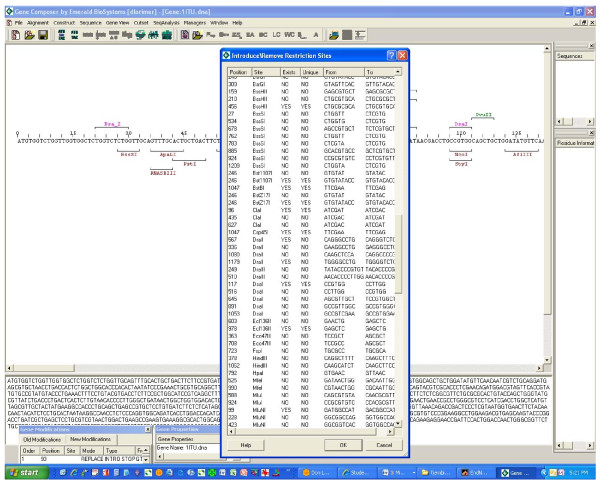
**Synonymous introduction and removal of DNA restriction enzyme sites**. The Protein-to-DNA algorithm of Gene Composer has generated a novel back-translated gene sequence, and has identified existing restriction sites that either can be eliminated or introduced (listed above or below the nucleic acid sequence respectively) by synonymous mutation without violating any user defined constraints on codon usage or G:C% content. Unique, single cutting restriction sites are shown in green. The inset window shows a detailed list the location of each restriction site, its current sequence (from: column), and the proposed alternate synonymous sequence (to: column) required to silently introduce/remove the restriction site.

Many expression vector plasmids have multiple cloning sites (MCS) with several unique restriction enzyme sequences in a defined order which facilitates unidirectional cloning of gene sequences. In order to take full advantage of the utility of multiple cloning sites, careful synthetic gene design allows one to use synonymous nucleotide changes to "silently" eliminate undesired MCS restriction endonuclease sites from the body of the open reading frame (Figure 6). In this way, the synthetic gene sequence can then be easily moved between vectors with common MCS restriction sites.

The degeneracy of the genetic code, even when constrained to the preferred codons of highly expressed proteins, usually allows sufficient flexibility to enable the "silent" introduction of unique restriction sites located at strategic positions within the open reading frame of the gene of interest. The introduction of unique restriction sites throughout the body of a gene enables it to be cleaved into several defined pieces, which may be designed to encode alternate mutations within each gene segment. In this way, re-ligation of the pools of mutant gene segments allows for the rapid generation of several combined mutations. This strategy can be used to select for protein variants with desired properties and can be a powerful combinatorial protein engineering strategy. It should be noted that within the within the restriction site database manager, users can specify any DNA sequence, including wild card sequences that they wish to consider for silent introduction or removal without altering the amino acid coding sequence.

#### Silent Removal of Cryptic Translation Initiation Sequences

When designing synthetic gene sequences for optimal protein expression it is important to consider that simple back-translation process may inadvertently design open reading frame sequences with one or more cryptic Shine-Dalgarno [22] or Kozak [23] translation initiation sites in any of the three possible reading frames [24]. The translation initiation signal for prokaryotic organisms (bacteria), was identified by Shine and Dalgarno [22], as a degenerate purine rich sequence with a general consensus of 5'-UAAGGAGGU-3' located ~7 nucleotides immediately upstream (on the 5' side) of a methionine codon (AUG). Given the bipartite nature of the Shine-Dalgarno initiator sequence and its relatively large size, it is usually a simple task to for Gene Composer to identify one or more synonymous single base changes that will effectively eliminate the undesired "cryptic" Shine-Dalgarno sequence within the constraints of previously defined codon usage frequencies. The Gene Design Module allows the user to choose one or more possible silent changes that will eliminate "cryptic" Shine-Dalgarno sequences.

In eukaryotic organisms, the Kozak consensus sequence for translation initiation is loosely defined as 5'GCCACCAUGG3' wherein translation initiation usually starts at a methionine AUG codon within the sequence. Given the relatively small size of the Kozak consensus sequence, there are relatively few possible alternative synonymous sequence choices that would eliminate an unwanted cryptic Kozak site. As such, Gene Composer has the flexibility to allow the user to define any unwanted sequence of any size. The software will choose any synonymous change that effectively eliminates the defined sequence, even if its use would be counter to previously defined codon usage parameters.

#### Silent Removal of Nucleic Acid and Codon Repeats

Sequence repeats of greater than five nucleotides in length are a source of ribosomal frameshifting, wherein the mRNA can "slip" out of frame [25]. Therefore, to improve translation fidelity, nucleotide repeats in mRNA should be silently eliminated by single base sequence changes to effectively destroy their "slippery" nature. Sequence repeats can be identified by the Gene Design Module and removed by silent mutation. This is achieved by simply replacing the first complete degenerate codon within any 5 base sequence repeat with an alternative synonymous codon regardless of any previously defined codon usage constraints. Repeated use of the same codon for a repeated amino acid (i.e. a hexa-histidine) has been reported to have a negative effect on translation kinetics and fidelity, likely due to depletion of charged tRNA pools [26-28]. As such, Gene Composer allows the user to identify any pair of duplicated degenerate codons, and replace either the first or second codon with an alternative synonymous codon.

#### Silent Introduction of Hidden "Ambush" Stop Codons in Alternate Reading Frames

As noted above, the ribosomal machinery is not perfect and can suffer a reading frame shift under certain circumstances, such as when it encounters a stable RNA hairpin, pseudoknot structure, or a "slippery" repeated nucleotide sequence [18]. Once a frameshift occurs, the resulting polypeptide will be synthesized with an out of frame encoded C-terminus until a stop codon is reached. In order to minimize the mis-translation distance traveled by a frameshifted ribosome, it is desirable to silently engineer hidden "ambush" stop codons into both the -1 (second) and +1 (third) reading frames [20]. The presence of ambush stop codons scattered throughout the gene helps to ensure that any frameshifted ribosomes will reach a termination codon sooner rather than later, thereby spending less overall time and energy wasted on translation of defective proteins. The degeneracy of the genetic code is highly accommodative of a wide range of frequencies of hidden stop codons, and genomic open reading frames appear to have an overabundance of "ambush" codons compared to gene sequences built solely on the basis of codon usage frequencies, suggesting that the genetic code has evolved to make use of "ambush" codons in some way (personal observation). Gene Composer allows users to engineer as many possible ambush codons as they may desire within the previously defined constraints of codon usage, G:C%, or local ΔG parameters. Generally, the introduction of an ambush stop codon every ~100 base pairs is sufficient to halt 2^nd ^or 3^rd ^frame translation and thereby promote the termination of any mistranslated proteins.

#### Silent Removal of Endoribonuclease Sites

Protein production levels from synthetic genes can sometimes be improved by synonymous codon engineering of mRNAs to resist destruction through the silent elimination of RNase cleavage sites. This is handled by Gene Composer using the same procedure described above for the elimination of cryptic Kozak sequences. Similarly, synonymous codon engineering by Gene Composer can be used to create "ACA-less" mRNAs for "single protein" production in strains of *E. coli *that carry an inducible MazF gene. This gene encodes the MazF endoribonuclease which cleaves any unstructured single-stranded RNA molecules that contain the "ACA" sequence [29]. Since most mRNA molecules naturally contain at least one ACA sequence, the inducible production of MazF in cells causes the destruction of all mRNAs, except those that lack the "ACA" sequence. With this approach, MazF is used to convert the *E. coli *cells into a "quasi-dormant" state, lacking all mRNA except for the mRNA encoded by the synthetic "ACA-less" gene. The cells in this growth arrested state can be concentrated and added to medium containing spin-labeled organic salts, which results in the NMR-ready labeling of only the protein of interest that is encoded by the synthetic "ACA-less" gene. This MazF procedure for producing only one protein of interest *in vivo *requires that the gene of interest be engineered to silently eliminate all "ACA" nucleotide combinations. Conveniently, the degeneracy of the genetic code allows all possible ACA coding sequences to be changed to alternative synonymous coding sequences. Gene Composer is capable of designing ACA-less genes through synonymous codon engineering which eliminates the ACA triplet in all three reading frames.

### Synthetic Gene Production

Once a codon engineered gene sequence has been formulated with the aid of the Gene Composer protein-to-DNA algorithm, the gene can either be purchased from a synthetic gene vendor, or be manufactured by PCR in a standard molecular biology lab from the Oligo-sets designed by the Gene Composer Gene Design Module. This module includes a gene-to-oligonucleotide algorithm that is used to plan all required overlapping oligonucleotides needed to synthesize a desired gene sequence by PCR [30-34], and for physically cloning gene derivatives into selected vectors by the most popular subcloning strategies including ligase independent cloning (LIC), recombinational cloning (ie. Gateway^®^), traditional restriction enzyme cloning, and the polymerase incomplete primer extension cloning method (PIPE) [14].

#### Oligo-set Creation

**Figure 7:**
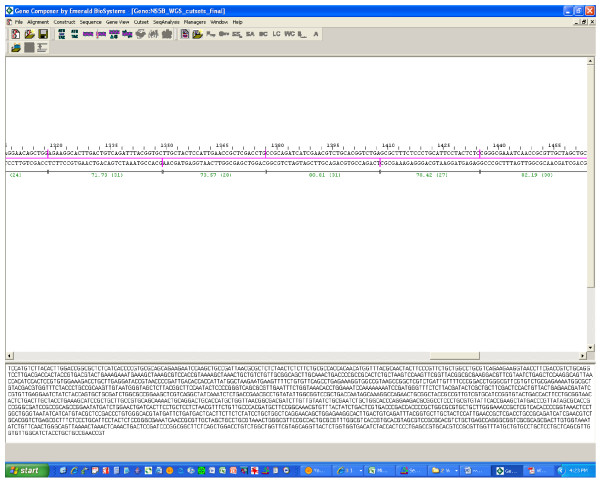
**Oligo-set generation**. The gene-to-oligonucleotide algorithm of Gene Composer has designed a set of overlapping oligonucleotides (Oligo-set) which can be used in pooled PCR reactions to assemble the gene synthetically. The top window shows all of the overlapping regions of oligonucleotides with annotation for the overlap T_m _and overlap base length in parentheses. The entire gene sequence is displayed in the lower panel.

Gene Composer has the ability to design overlapping oligonucleotide sets (Oligo-sets) for a variety of reported PCR gene assembly methods that use a range of different oligonucleotide building block sizes (Figure 7). As such, the Oligo-set creation feature of the gene-to-oligo algorithm requires five user defined oligonucleotide parameters: i) a maximum oligo size, ii) a minimum oligo size, iii) a target overlap size between any top and bottom strand oligos, iv) the minimum Gibbs free energy (ΔG) allowable for intramolecular oligonucleotide folding, and iv) the salt concentration present in the PCR reactions. The first three parameters are checked to ensure that they do not conflict with the following rules. The minimum must be less than the maximum, and the minimum oligo size must be greater than or equal to twice the overlap size. The difference between maximum and minimum oligo sizes must be greater than 5.

With these parameters defined, the gene-to-oligo algorithm computationally cuts the top and bottom strand sequences at random positions that are otherwise constrained only by the aforementioned oligo size and overlap constraints. The algorithm starts on the top strand and generates the first cut by choosing a random integer value between the maximum and minimum sizes (inclusive). The algorithm continues marching down the top strand in the 5' to 3' direction generating cuts until the remaining length of the sequence is less than the minimum oligo length. Once this point is reached, the algorithm iteratively shifts all upstream cut sites by -1 base until the terminal oligo length is the size of the target overlap length. This process adjusts the cuts across the top strand such that the final ensemble of cuts produces oligos that meet all of the user defined size parameters. Once the top strand has been cut up, the algorithm then generates bottom strand cuts by making calculated cuts at nucleotide positions approximately half way between the ends of the top strand oligos. This ensures that each bottom strand cut falls in the approximate middle of two top-strand oligos, which is validated in a final step that checks that the top and bottom strand oligo sizes and overlaps conform to the user defined parameters.

The gene-to-oligo algorithm is run thousands of times to generate a large number of candidate Oligo-sets from which to choose. The Oligo-sets are then ranked according to their calculated average melting temperature (T_m_) for the overlapping segments of each possible top and bottom strand pair [35]. The ideal Oligo-set is one that has the highest average T_m _value and the lowest overall variance in T_m_, which together help ensure fidelity in oligonucleotide pairing during pooled PCR reactions for gene assembly. Moreover, an Oligo-set should not contain any oligonucleotides that can form stable hairpins (palindromic sequences), or which have the same or similar sequence as other oligonucleotide members. Therefore, the gene-to-oligo algorithm selects only the best candidate Oligo-set which has the highest average T_m_, lowest variance in T_m_, and have a calculated ΔG for intramolecular folding which is above the value set during the backtranslation step [17].

Once the top candidate Oligo-set has been selected, the gene-to-oligo algorithm proceeds to make further improvements to the Oligo-set by adjusting the oligonucleotide end points for any top and bottom strand oligo pairs whose overlap T_m _values are furthest from the mean (both above and below). Once identified, the algorithm determines how it can shift the oligonucleotide endpoints to either raise the overlap T_m _and/or bring it closer to the mean. With each shift, a check is done to ensure that the Oligo-set variables (minimum/maximum size, overlap size) are not violated, and that the T_m _is closer to the mean and the overall T_m _variance has been reduced. The final Oligo-set is checked again to ensure that no oligonucleotides violate the minimal allowable ΔG for intramolecular folding.

#### Gene Synthesis by PCR Assembly in Combination With Base Mismatch Surveillance

**Figure 8:**
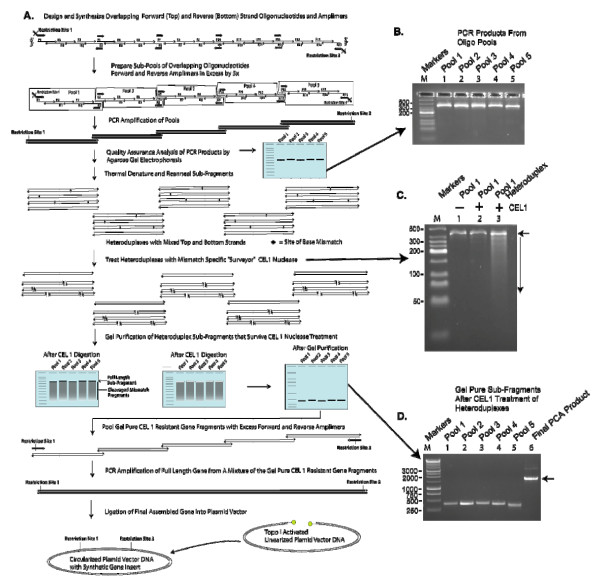
**PCA assembly of a gene from overlapping Oligo-sets**. **A**, A schematic diagram of the PCR assembly of a synthetic gene is shown (NS5B, HCV Polymerase). After design of the gene and creation of an Oligo-set using Gene Composer, the PCR gene synthesis protocol is initiated by pooling overlapping complementary oligonucleotides which together comprise individual sub-fragments (Pools 1–5) of the gene which are amplified by PCR and analyzed by agarose gel electrophoresis. **B**, PCR products of sub-fragments from oligo Pools 1–5. Each product band is about 400 bp. **C**, The amplification products of Pool 1 (lane 1) are denatured and re-annealed to generate heteroduplexes which are treated with CEL1 nuclease to digest any DNAs with base-mismatches (lane 3, Pool 1 shows a smear of DNAs below ~400bp indicating that CEL1 was able to digest some of the heteroduplexes, in comparison to lane 2 which is CEL1 treated Pool 1 that was not denatured and re-annealed and could therefore not be digested with CEL1). **D**, The remaining CEL1 resistant intact (undigested) heteroduplex sub-fragments are gel purified (Pools 1–5, lanes 1–5) and pooled for final assembly by PCR (lane 6, arrow). The expected size of the full length gene is about 2,000 bp, and this material can be cloned directly into Topo activated vectors (Invitrogen).

The Gene Composer protocol for PCR assembly (PCA) of synthetic genes from designed oligonucleotides (Oligo-sets) is illustrated in Figure 8 and is based on an amalgam of the best practices in previously described PCA methods [30-34]. PCA methods involving pools of large numbers of oligonucleotides often fail to produce the desired full-length gene product, which can usually be attributed to the production of mis-primed PCR products [33], which are more likely to form as the variance in the T_m _of overlaps between top and bottom strands increases. The variance in overlap T_m _is influenced by both the number of oligos in the pool and their average overlap length. Therefore, in order to improve the successful outcome of gene synthesis, our Gene Composer PCA protocol starts with the PCR production of gene fragments from sub-pools of oligos. Once the successful production of each overlapping gene fragment has been confirmed by agarose gel electrophoresis or capillary electrophoresis, all sub-gene fragments are pooled for PCR amplification of the full length gene using an excess of flanking primers covering the extreme 5' and 3' ends of the desired gene. While it is certainly possible to obtain complete genes by PCA from a large pool of oligos, we have found that the failure rate for PCA from large pools is too high to justify this as a standard approach to gene synthesis, and have therefore adopted the sub-fragment/pool approach to PCA for synthetic genes.

Most PCA-based gene synthesis methods can suffer from a mutation rate of ~2 to 4 point mutations per 1–2 Kbp of synthetic gene product [4,36-38]. The majority of synthetic gene mutations are single nucleotide deletions, and to a much lesser extent, base changes. The single nucleotide deletions are the result of the presence of "N-minus-1-mers" as contaminants in preparations of synthetic oligonucleotides while the base changes are usually attributable to polymerase mistakes that occur during PCR assembly. N-minus-1-mers are oligonucleotides that are missing one or more base(s) somewhere within the sequence, which are the result of unavoidable ~<1.0% inefficiencies in each round of solid phase oligonucleotide synthesis [4]. In order to eliminate base deletions and mistakes in PCA-mediated gene synthesis, researchers have taken advantage of the ability of mismatch specific endonucleases to specifically recognize and cleave both DNA strands at the site of base pair mismatches in heteroduplex DNA. This editing procedure involves the thermal denaturation and re-annealing of full-length PCR gene products to produce heteroduplexes. During the denaturation and re-annealing process, the chance that two strands of any given duplex will re-anneal to each other is extremely low. As such, only hetero-duplexes form upon re-annealing, wherein they are comprised of strands that originated from two different duplexes. Heteroduplexes that lack parity due to a mistake in either the top or bottom strands will have a base pair mismatch which can be recognized and cleaved by a mismatch specific endonuclease. After digestion of the mismatched heteroduplexes, the accurately synthesized full-length DNA molecules can be isolated away from the cleaved mutants by agarose gel purification. Mismatch-specific endonucleases include S1 nuclease, T4 endonuclease VII, T7 endonuclease I, CEL 1 [30], and the MutHLS proteins. These enzymes represent efficient tools for greatly improving the accuracy of gene synthesis.

The use of mismatch specific endonuclease surveillance in conjunction with PCA greatly reduces the error rate synthetic gene production. Nevertheless, in order to obtain a final sequence validated synthetic gene of ~1–2 Kbp, one may have to examine several (~5) independent gene clones to identify one which is 100% accurate. Single base mistakes can also be repaired by site-directed mutagenesis, and/or by reconstructing the final gene from sub-cloned gene fragments that are free of mutations. Finally, many researchers may simply wish to purchase a fully sequence validated synthetic gene of their own design from a number of synthetic gene vendors.

#### Examples of PCA Gene Synthesis

We chose three genes as examples of our methods from design to final synthetic gene product. These are the human protein kinase P38α, the bacterial protein FtsZ from *Bacillus subtilis*, and the Hepatitis C viral polymerase NS5B. The sequences of these genes were re-designed by codon engineering using Gene Composer as described in the Materials and Methods. In Figure 8 we demonstrate the stepwise results from each stage in the synthetic gene production process by PCA with mismatch specific endonuclease CEL1 error correction, using the re-designed NS5B gene as an example. In addition to constructing the re-designed genes, we also assembled their native cDNA counterparts by PCA from overlapping Oligo-sets planned by Gene Composer.

The sequence analysis results for several clone isolates of the six synthetic genes (native cDNA and Gene Composer designs for P38α, FtsZ, and NS5B) are presented in Table 1. The most common sequence error observed in the products of PCA gene synthesis are single base deletions which (despite the use of mismatch endonuclease error removal) most likely have their origins rooted in the N-minus-1-mer problem with oligonucleotide synthesis wherein despite high efficiency in oligonucleotide synthesis there is a very small (but nonetheless unavoidable) population of oligonucleotides missing one base somewhere within their sequence. However, even with the relative preponderance of single base deletions, the statistics suggest that for genes less than ~2 Kbp, choosing 4 clones to sequence will likely yield at least one correct clone.

**Table 1:** Sequence errors for 6 synthesized genes.

**Gene**	**Correct Clones**	**Synonymous Changes**	**Non-Synonymous Changes**	**Deletions**	**Other Mutations**	**Totals**
**FtsZ**	4	1	3	16	0	24

**FtsZ/cDNA**	2	1	2	14	5	24

**NS5B**	6	1	0	9	8	24

**NS5B/cDNA**	4	0	0	7	6	17

**P38**	9	2	4	6	0	21

**P38/cDNA**	8	1	0	3	0	12

**Totals**	33 (27%)	6 (5%)	9 (7%)	55 (45%)	19 (16%)	122

Multiple clones representing each of the 6 synthesized genes described in this manuscript were sequenced and the types or errors tabulated. On average about 25% of the sequenced clones were correct. Errors resulting from base deletions were seen in about 50% of clones examined, most likely the result of N-minus-1-mer problem with oligonucleotide synthesis wherein despite high efficiency in oligonucleotide synthesis there is a very small amount but nonetheless unavoidable population of oligonucleotides missing one base somewhere within their sequence [4].

## Discussion

Whole gene synthesis is rapidly becoming a powerful and cost effective technology for creating novel proteins and improving protein expression. To capitalize on the availability of low cost synthetic genes, we have created a database software package called **Gene Composer™ **that facilitates the information-rich design of protein constructs and their nucleic acid coding sequences. This user friendly software package enables the facile design of totally novel nucleic acid sequences which are codon engineered for improved protein production in heterologous expression systems. We believe that computer aided design software will allow researchers to efficiently design protein constructs and synthetic genes which they can order according to their own design (not just the design provided by any given synthetic gene vendor). The prospect of increasingly affordable synthetic genes opens the opportunity to explore the effects of synonymous gene sequence engineering for improved protein production, or in some cases reduced protein production [39]. Our efforts in building Gene Composer for synthetic gene design are geared towards the development of software that allows the user to have total control over numerous gene design parameters and to have the software be responsible for planning out all of the required DNA (oligos, PCR products, vectors) manipulations to be handled by liquid handling robots. Gene Composer is not just a design tool but also an informatics engine for manufacturing constructs. In this way, researchers can spend more time designing improved constructs, and less time having to think about how to make the construct.

Another goal in developing Gene Composer is to aid the dissemination of synthetic DNA technology to even very small labs with modest investments in recombinant DNA equipment (PC computer, thermal cycler, agarose gel electrophoresis, UV illumination box, microcentrifuge, and temperature controlled environments). We have shown that our PCA method for synthetic gene assembly from Oligo-sets defined by Gene Composer works reliably for both highly engineered synthetic gene sequences and native cDNA sequences, with reasonably low error rates of 1–2 mutations per 2 Kbp. Thus, Gene Composer can aid even the smallest lab to produce synthetic genes in any location where oligonucleotides can be made or ordered for delivery.

Several companies now offer synthetic gene production services (DNA 2.0, ; Codon Devices, ; Blue Heron, ; Geneart, ; BioBasic, ; GenScript, ; Top Gene Technologies, , and others) and competitive technology development is expected to continually drive prices lower. In general, most synthetic gene production companies also offer limited gene design services, for example to design genes with preferred codons of a selected expression system. However, we anticipate that the design parameters for improved protein production through synonymous gene sequence engineering of open reading frames will ultimately require a more sophisticated user driven approach. For this reason, we have intentionally enabled Gene Composer to accept numerous user defined settings for gene design parameters.

## Conclusion

For structural biology applications, protein construct engineering is guided by comparative sequence and structural information, which allows the researcher to better define domain boundaries for terminal deletions and non-conserved regions for surface mutants. As such, our efforts in developing Gene Composer software are to integrate protein construct design together with synthetic gene design where researchers are allowed flexibility to design genes according to specific user-defined parameters. Table 2 offers an analysis of Gene Composer features, relative to other available tools [38,40-43]. The software allows users to design genes such that numerous parameters can be held constant or varied. This should allow users the opportunity to carryout comparative protein expression studies with designed genes such that the design impact of one or more variables can be carefully measured. We do anticipate that such studies may require hundreds of different experiments to identify the value of any given design parameter for improved protein production. Moreover, we anticipate that the optimal design parameters may be specific for certain protein families and expression systems. For example, codon engineering may be used to alter translation kinetics with respect to protein domain organization, which in turn could modulate protein function [40]. Thus, the burgeoning field of synthetic biology has great promise to benefit from gene design tools such as Gene Composer. In the sister manuscript to this article [44] we demonstrate that the combination of construct and codon engineering can have great utility for improved heterologous protein production of infectious disease targets for structural studies.

**Table 2:** Software and Website Applications for Designing Synthetic Genes.

**Name**	**Database**	**Codon Engineering**	**Sequence Engineering**	**Oligo-nucleotide Engineering**	**Construct/Vector Engineering**	**Protein Engineering**
**Gene Composer**	• CUT	• CWeighted Random	• Restriction Sites	• Gene Synthesis	• **Yes**	• **Yes**
	• Genes	• G:C Content	• mRNA structure	• Mutagenesis		
	• Constructs	• Custom	• Repeat Removal			
	• Alignments		• Ambush Stop Codon			
			• User defined Sequences			

**Optimizer**	• CUT	• One Amino Acid – one Codon	• **No**	• **No**	• **No**	• **No**
		• Guided Random				
		• Custom				

**Gene Designer**	• **No**	• One Amino Acid – One Codon	• Restriction Sites	• **No**	• **No**	• **No**
		• Random	• Repeat Removal			

**Gene Optimizer**	• **No**	• Multiple parameter	• mRNA structure	• **No**	• **No**	• **No**
			• Repeat Removal			
			• mRNA Splice Sites			

**DNA Works**	• **No**	• Random	• Restriction Sites	• Gene Synthesis	• **No**	• **No**

**Database **refers to the presence or absence of a database to store codon usage tables (CUT), gene sequences (Genes), complete plasmid construct sequences (Constructs), and alignments of protein sequences (Alignments). **Codon Engineering **refers to the methods used for back-translation of amino acid sequences into nucleic acid sequences. **Sequence Engineering **refers to the ability to alter the nucleic acid sequence to introduce or remove restriction sites, remove sequence repeats, alter potential mRNA secondary structures, remove mRNA splice sites or any other user defined sequence. **Oligonucleotide Engineering **refers to the ability to design oligonucleotides to for gene synthesis or to create mutagenic primers. **Construct/Vector Engineering **refers to the ability to visualize and create complete plasmid expression vectors using designed gene sequences. **Protein Engineering **refers to the ability to create protein alignments to design specific protein constructs. Gene Composer: Emerald Biosystems, ; Optimizer: Universitat Rovira i Virgili, genomes.urv.es/OPTIMIZER/; Gene Designer: DNA2.0, ; Gene Optimizer: GeneArt, ; DNA Works: Helix Systems, .

## Methods

### Reagents

Oligonucleotides were supplied by IDT (Coralville, IA) as standard, desalted oligos and were used without further purification. KOD polymerase was obtained from Novagen (Madison, WI), and used for all PCR reactions following the manufacturer's instructions. PCR fragments were gel purified from agarose gels using a Qiagen Gel Extraction Kit (Qiagen, Valencia, CA). Plasmid DNA was prepared using a Qiagen Plasmid Purification Kit (Qiagen, Valencia, CA). Restriction enzymes were obtained from Fermentas (Hanover, MD) or New England BioLabs (Ipswich, MA). T4 DNA ligase was purchased from New England Biolabs. CEL1 nuclease was purchased from Transgenomic, Inc (Omaha, NE).

### Gene design and Gene Composer settings

Synthetic DNAs encoding FtsZ (Genbank code NP_389412, amino acids 1–382), P38α (NP_620581, amino acids 1–360) and Hepatitis C viral NS5B (AAK08509, amino acids 2420–2898) were engineered for expression in *E. coli *using Gene Composer software (, Emerald BioSystems, Bainbridge Island, WA). Back-translation of the amino acid sequence was accomplished using an *E. coli *highly expressed protein codon usage table (Table 3). A minimum threshold cut off for codon usage frequency was set at 2%, eliminating codons with usage frequencies less than 2%. Using these settings every amino acid, except Met and Trp, have at least 2 codons available for use.

**Table 3:** Codon usage frequency table for optimal expression in *E. coli*.

**AA**	**Codon**	**Freq**	**AA**	**Codon**	**Freq**
	
**Ala**	GCA	0.28	**Leu**	CTT	0.05
**Ala**	GCC	0.07	**Leu**	TTA	0.03
**Ala**	GCG	0.21	**Leu**	TTG	0.02
**Ala**	GCT	0.45	**Lys**	AAA	0.81
**Arg**	AGA	0.02	**Lys**	AAG	0.19
**(Arg)**	**AGG**	**0**	**Met**	ATG	1
**(Arg)**	**CGA**	**0**	**Phe**	TTC	0.79
**Arg**	CGC	0.24	**Phe**	TTT	0.21
**(Arg)**	**CGG**	**0.01**	**Pro**	CCA	0.08
**Arg**	CGT	0.73	**(Pro)**	**CCC**	**0.01**
**Asn**	AAC	0.91	**Pro**	CCG	0.82
**Asn**	AAT	0.09	**Pro**	CCT	0.08
**Asp**	GAC	0.72	**Ser**	AGC	0.15
**Asp**	GAT	0.28	**(Ser)**	**AGT**	**0.01**
**Cys**	TGC	0.8	**Ser**	TCA	0.02
**Cys**	TGT	0.2	**Ser**	TCC	0.39
**Gln**	CAA	0.14	**Ser**	TCG	0.04
**Gln**	CAG	0.86	**Ser**	TCT	0.39
**Glu**	GAA	0.83	**Stop**	TAA	0.83
**Glu**	GAG	0.17	**Stop**	TAG	0.17
**(Gly)**	**GGA**	**0**	**Stop**	TGA	0
**Gly**	GGC	0.5	**Thr**	ACA	0.02
**(Gly)**	**GGG**	**0.01**	**Thr**	ACC	0.56
**Gly**	GGT	0.48	**Thr**	ACG	0.05
**His**	CAC	0.83	**Thr**	ACT	0.36
**His**	CAT	0.17	**Trp**	TGG	1
**Ile**	ATA	0.02	**Tyr**	TAC	0.8
**Ile**	ATC	0.86	**Tyr**	TAT	0.2
**Ile**	ATT	0.12	**Val**	GTA	0.21
**(Leu)**	**CTA**	**0.01**	**Val**	GTC	0.07
**Leu**	CTC	0.06	**Val**	GTG	0.15
**Leu**	CTG	0.83	**Val**	GTT	0.57

Frequency refers to the percentage occurrence of synonymous codons encoding amino acids in *E. coli *highly expressed proteins. The nucleic acid sequences of the following genes were combined into a single pseudo-gene and then used in the Kazusa Countcodon program  with eubacterial translation exceptions to generate a codon usage table for that pseudo-gene: ompA (V00307), atpE (V00266), cybB (AP009048), cybC (U14003), sdhC (AP009048), groEL (AP009048), tufA (AP009048), rpsA (AP009048), rpsB (NC_000913), rpoA (AP009048), rpoB (AP009048), rpoC (AP009048), pheS (AP009048), pheT (AP009048), lysS (AP009048). Codons in parentheses fall below the 2% frequency cutoff, and are not used during back-translation when the threshold for codon usage is set at 2%.

Native cDNAs encoding these proteins were also assembled (FtsZ, M22630, bp 1939–3084; P38α, NM_139012, bp 339–1497; NS5B, AF333324, bp 7599–9371). No sequence optimization or alteration was carried out for the cDNAs except to exclude certain restriction enzyme sites from the body of the open reading frame by synonymous codon engineering so as to facilitate cloning into the expression vectors.

Synthetic genes were designed using the Gene Composer protein-to-DNA back-translation algorithm using the *E. coli *highly expressed protein codon usage table together with the "ΔG" feature engaged to ensure that the resulting mRNA sequence would not contain any highly stable local mRNA folding elements. The minimal codon usage cut off was set at 2% and the ΔG optimization was set to -15 to -25 kcal/mol. In the ΔG mode, products of the back-translation are interrogated for the presence of stable mRNA hairpins in 50 bp windows. The process starts at base pair 1 and examines the sequence from base pairs 1 to 50 then shifts 10 bp and interrogates again until the entire length of the gene has been examined. Back translated products with hairpins more stable than the desired settings were rejected. Once a sequence meeting the codon usage and ΔG cut-off was generated, other sequence specific changes were made as follows. To prevent the accumulation of mis-translated proteins arising from ribosome slippage, improper translation initiation or sequence errors, we introduced "ambush" stop codons in the 2^nd ^and 3^rd ^reading frames approximately every 100 base pairs by substitution of synonymous codons. For example, the sequence ACA AGA CAC encoding the amino acid sequence Thr-Arg-Gln can be changed to ACT AGA CAC, preserving the amino acid sequence but introducing a stop codon (TAG) in the third reading frame. Cryptic Shine-Dalgarno sequences upstream from any AUG in all three reading frames were also removed to prevent improper cryptic translation initiation. Restriction enzyme recognition sites for BamHI and HindIII were also silently removed by synonymous codon changes from both the synthetic genes and native cDNAs to facilitate cloning.

The designed genes were then converted into Oligo-sets using the Gene Composer gene-to-oligo algorithm. The overlapping oligonucleotides on top and bottom strands of the NS5B gene were optimized for overlapT_m _(65.84 to 87.84°C), ΔG of oligo folding (-1.50 to -17.80 kcal/mol), and length (50–70 bases). The "Create Primers" algorithm was used to organize the overlapping oligonucleotides into pools of 5 or 6 with combined segment lengths of 300–500 bp for each pool. The flanking amplification primers were set to a T_m _of 65°C to 80°C and a length of 20 to 30 bases. Restriction sites were added as 5' and 3' flanking sequences to the ends of the primers in the primer design tool of Gene Composer.

The native gene sequences were divided into Oligo-sets using the manual Oligo-set creation feature in Gene Composer. The oligo size range was set to 50–70 bases and the overlap to 23–40 bases. The specific Oligo-set used was chosen from a calculated list of more than 100 possible Oligo-sets and was based on high minimum T_m _and low T_m _variance for overlapping segments of the oligonucleotides. The autoimprove T_m _and improve ΔG features were omitted from the Oligo-set optimization for cDNA samples to prevent the introduction of synonymous changes. Primers were generated in the same manner and with the same parameters as for synthetic genes. The Oligo-sets for cDNA gene synthesis were ordered from the same vendors and assembled by PCA using the same methods as for the synthetic codon engineered genes.

### PCA gene synthesis

A two-stage PCR method is used to assemble synthetic and cDNA coding sequences with an intervening mismatch-dependent-endonuclease treatment of PCR products to reduce errors arising from PCR or from oligonucleotides containing synthesis mistakes.

In Step 1, defined pools of overlapping oligonucleotides and PCR primers (Oligo-sets) were used in individual PCR reactions to produce individual gene sub-fragments of 400–500 bp. Together, each of the gene sub-fragments span the entire gene coding sequence, with overlaps between sub-fragment ends of ~20–30 base pairs. Target genes were parsed by Gene Composer into pools (Oligo-sets) of 10–12 overlapping oligonucleotides (5–6 oligo pairs) for PCR amplification. Oligos in a solution containing 2.8 mM Tris-HCl, pH 7.6, 0.4 mM KCl, 0.04 mM dithiothreitol, and 50 mM NaCl were mixed in an equimolar ratio at a final concentration of 4 μM each. The mixture was then diluted 16-fold with water to create a working stock for PCR, of which 2 μl of each pool were individually amplified in a 50 μl PCR reaction using KOD polymerase (2.5 Units) without amplification primers in the manufacturer's buffer containing 0.12 M Tris-HCl, pH 8.0, 10 mM KCl, 6 mM (NH_4_)_2_SO_4_, 0.1% Triton X-100, 0.001% BSA. Four cycles of PCR were performed at 94°C for 30 seconds, 72°C for 90 seconds, and 72°C for 45 seconds. After 4 cycles, forward and reverse amplification primers were added to a final concentration of 200 nM each, and the reactions were amplified for 26 cycles of 94°C for 30 seconds, 68°C for 90 seconds, and 72°C for 45 seconds, followed by a final extension of 10 minutes at 72°C. Reaction products were then separated on an agarose gel and the excised bands of appropriate size were purified using a standard gel extraction kit (Qiagen) into a volume of 50 μl of buffer (10 mM Tris-HCl, pH 8.5).

In Step 2, the gel purified sub-fragments from Step 1 were heat denatured at 95°C for 10 minutes (20 μl at 50 ng/μl), and then reannealed to form heteroduplexes by decreasing the temperature of the solution at a rate of 0.1 degree per second for 10 minutes in a thermocycler in a buffer containing 100 mM Tris-HCl, pH 8.0, 1 mM EDTA and 1 M NaCl. The heteroduplexes were then treated with the mis-match specific endonuclease CEL1 nuclease (5 μl, Transgenomic, Inc.) in a 50 μl reaction at 40°C for 60 minutes (10 mM Tris-HCl, pH 8.8, 75 mM KCl, 1.5 mM MgSO_4_). The digested products were separated by agarose gel electrophoresis and the "error-free" CEL1 resistant bands were purified using a standard gel extraction kit (Qiagen) producing a final volume of 50 μl of purified CEL1 nuclease resistant sub-fragment in 10 mM Tris-HCl, pH8.5.

In Step 3, the full length coding sequences were assembled using PCR by pooling 2 μl (~1 μg) of each of the gel purified CEL1 resistant sub-fragments in a 50 μl reaction using KOD polymerase (2.5 Units) (Novagen, Madison, WI) in the manufacturers buffer (0.12 M Tris-HCl pH 8.0, 10 mM KCl, 6 mM (NH_4_)_2_SO_4_, 0.1% Triton X-100, 0.001% BSA containing 200 nM of the extreme 5' and 3'amplification primers (designed by Gene Composer) containing the terminal restriction enzyme recognition sites (BamHI and HindIII) or other sequences necessary for final construct cloning. PCR was performed for 26 cycles of 94°C for 30 seconds, 68°C for 90 seconds, and 72°C for 45 seconds, followed by a final extension of 10 minutes at 72°C. The final PCR products were then gel purified (with or without prior restriction enzyme digestion), and cloned into the desired expression vectors. Alternatively, the final PCR products can be subjected directly to LIC or PIPE cloning methods.

### Cloning

The concentration of gel purified synthetic genes from PCA was determined by absorbance at 260 nm using a NanoDrop ND-1000 spectrophotometer (Thermo Scientific, Waltham, MA). DNA concentrations were adjusted to 50 ng/μl or greater in 10 mM Tris-HCl, pH 8.5 and then used for cloning into the pCR-Blunt II-TOPO vector (Invitrogen) according to the manufacturer's instructions. One Shot™ TOP10 chemically competent *E. coli *(Invitrogen) were transformed with 4.5 μl of the cloning reaction. Aliquots of 50 μl and 200 μl of transformed bacteria culture were spread on 2xYT agar plates containing 50 μg/ml kanamycin and incubated overnight at 37°C. Single colonies of each of the constructs were grown overnight in 2xYT media containing kanamycin (30 μg/ml) and plasmid DNA was isolated by alkaline lysis using the Qiagen plasmid purification kit according to manufacturer specifications. Clones were sequenced with vector primers M13F(-21) (5'-TGTAAAACGACGGCCAGT-3') and M13R(-29) (5'CAGGAAACAGCTATGACC-3') by dideoxy termination using an Applied BioSystems, Inc. (Foster City, CA) 3730 capillary electrophoresis instrument. Sequence analysis was carried out using Sequencher 4.7 DNA Sequence Analysis Software (GeneCodes, Ann Arbor, MI). One correct clone for each of the constructs was selected and digested from the pCR-Blunt II-TOPO vector via double digest using BamHI and HindIII restriction enzymes (Fermentas) according to manufacturer specifications. Digested products were gel purified and the DNA was quantified by absorbance at 260 nm using a NanoDrop ND-1000 spectrophotometer. A 3:1 molar ratio of the insert to vector (15 ng/μl) is used to subclone into the final expression vector using T4 DNA ligase (New England Biolabs) following the manufacturer guidelines, but increasing the suggested ligation time from 10 minutes to 30 minutes in a 10 μl reaction at 22°C in buffer containing 50 mM Tris-HCl pH 7.5, 10 mM MgCl_2_, 10 mM dithiothreitol, 1 mM ATP. One Shot TOP10 Chemically Competent *E. coli *(Invitrogen) are transformed with 6 μl of the ligation reaction. Aliquots of 50 μl and 200 μl of transformed bacteria were spread on 2xYT plates containing 100 μg/ml ampicillin and incubated overnight at 37°C. Resulting colonies were screened for proper insert using PCR and vector specific primers and verified by DNA sequencing as described above.

### PIPE (Polymerase Incomplete Primer Extension) Cloning

The gene of interest is amplified in the insert PCR reaction (iPCR) with chimeric primers, with homology to both the gene terminus and the vector junction (see Figure 4 for details). The entirety of the expression vector into which the gene will be cloned is amplified in the vector PCR reaction (vPCR), with primers that exclude the *ccdB *negative selection cassette. PCR reactions are performed using pfuTurbo (Invitrogen). The extension time for the iPCR is 3 minutes, while the extension time for the vPCR is adjusted to 14 min. to allow sufficient time for amplification to occur. The vector for PIPE cloning is a derivative of pET28a into which the coding sequence of Smt3 has been cloned followed by a ccdB cassette. Equal parts crude iPCR product and crude vPCR product are combined and used to transform competent cells.

## Availability and requirements

Gene Composer™ software can be downloaded for free from  by following a simple click-through registration license. Operating System: Windows^® ^2000/XP Hardware: Pentium^® ^4 or Athlon^® ^at 1 GHz with 1 GB RAM

## Abbreviations

Oligo: Oligonucleotide; G:C%: guanine:cytosine percentage content; PCR: Polymerase chain reaction; LIC: Ligase independent cloning; PCA: PCR based gene assembly; MCS: multiple cloning sites; CUT: codon usage table; vPCR: PCR amplification of vector DNA for PIPE cloning; PIPE: Polymerase incomplete primer extension; iPCR: PCR amplification of insert DNA for PIPE cloning; NMR: nuclear magnetic resonance.

## Authors' contributions

DL contributed to software design, experimental strategy, analyzed data, and drafted the manuscript. AR contributed to experimental strategy, analyzed data, and drafted the manuscript. JW and MM wrote the *Gene Composer *database software package. EW, AB(1) and RG designed the gene sequences, designed the oligo-sets, performed the PCA gene assemblies and analyzed DNA sequences. AB(2) and LS conceptualized experimental strategy and guided the manuscript preparation. All authors read and approved the final manuscript.
